# Retraction for Abolnik, Complete Genome Sequence of *Mycoplasma meleagridis*, a Possible Emerging Pathogen in Chickens

**DOI:** 10.1128/genomeA.00695-15

**Published:** 2015-06-04

**Authors:** Celia Abolnik

**Affiliations:** Poultry Section, Department of Production Animal Studies, Faculty of Veterinary Science, University of Pretoria, Onderstepoort, South Africa

## RETRACTION

Volume 3, no. 3, e00380-15, 2015. The author hereby retracts this article.

The author was alerted that an American Type Culture Collection (ATCC) strain sequence for *Mycoplasma meleagridis* had recently been released. Phylogenetic analyses of the 16S rRNA gene and intergenic spacer region (IGSR) of all avian *Mycoplasma* spp., including the ATCC *Mycoplasma meleagridis* strain and strain B2096 8B, were conducted, and subsequently it was discovered that the latter is *Mycoplasma gallinaceum* and not *Mycoplasma meleagridis* as reported.

The author assumes all responsibility for this error and apologizes for any inconvenience caused to the readers of *Genome Announcements*. B2096 8B represents the first full genome sequence of *Mycoplasma gallinaceum*, and a revised manuscript is in preparation.

